# Implications of Bicuspid Aortic Valve Disease and Aortic Stenosis/Insufficiency as Risk Factors for Thoracic Aortic Aneurysm

**DOI:** 10.31083/j.rcm2406178

**Published:** 2023-06-19

**Authors:** Habib Jabagi, Dov Levine, Lara Gharibeh, Chiara Camillo, Estibaliz Castillero, Giovanni Ferrari, Hiroo Takayama, Juan B. Grau

**Affiliations:** ^1^Division of Cardiothoracic Surgery, The Valley Hospital, NJ 07450, USA; ^2^Department of Cardiovascular Surgery, Mt. Sinai Hospital, Icahn School of Medicine, New York, NY 10029, USA; ^3^Department of Surgery, Columbia University, New York, NY 10027, USA; ^4^Department of Biochemistry, Microbiology and Immunology, University of Ottawa, Ottawa, ON K1N 6N5, Canada; ^5^Division of Cardiac Surgery, University of Ottawa Heart Institute, Ottawa, ON K1Y 4W7, Canada

**Keywords:** bicuspid aortic valve, aortopathy, thoracic aortic aneurysm, aortic stenosis/regurgitation

## Abstract

Bicuspid Aortic Valves (BAV) are associated with an increased incidence of thoracic aortic aneurysms (TAA). TAA are a common aortic pathology characterized by enlargement of the aortic root and/or ascending aorta, and may become life threatening when left untreated. Typically occurring as the sole pathology in a patient, TAA are largely asymptomatic. However, in some instances, they are accompanied by aortic valve (AV) diseases: either congenital BAV or acquired in the form of Aortic Insufficiency (AI) or aortic stenosis (AS). When TAA are associated with aortic valve disease, determining an accurate and predictable prognosis becomes especially challenging. Patients with AV disease and concomitant TAA lack a widely accepted diagnostic approach, one that integrates our knowledge on aortic valve pathophysiology and encompasses multi-modality imaging approaches. This review summarizes the most recent scientific knowledge regarding the association between AV diseases (BAV, AI, AS) and ascending aortopathies (dilatation, aneurysm, and dissection). We aimed to pinpoint the gaps in monitoring practices and prediction of disease progression in TAA patients with concomitant AV disease. We propose that a morphological and functional analysis of the AV with multi-modality imaging should be included in aortic surveillance programs. This strategy would allow for improved risk stratification of these patients, and possibly new AV phenotypic-specific guidelines with more vigilant surveillance and earlier prophylactic surgery to improve patient outcomes.

## 1. Introduction

With an incidence of 7.6 per 100,000 persons, thoracic aortic aneurysms (TAA) are a common aortic pathology, and the 19th leading cause of death in the United States [1, 2, 3]. Traditionally defined as dilatation of the aorta to ≥1.5 times its normal diameter, TAA are largely asymptomatic and often diagnosed as incidental findings on unrelated routine imaging procedures. Over time, TAA can lead to adverse aortic events (AAE), which are often lethal complications such as dissection and rupture. Genetic predisposition, hypertension, hemodynamic forces, smoking, atherosclerosis, and pregnancy are all contributing risk factors of TAA pathophysiology [4]. While most TAA occur as isolated pathologies, they can develop as a consequence of aortic valve (AV) disease; either acquired Aortic Insufficiency (AI) and/or aortic stenosis (AS), or congenital, with the most common being Bicuspid Aortic Valves (BAV).

Aortic insufficiency, or regurgitation, occurs when AV integrity is compromised due to inadequate leaflet closure. Characterized by diastolic blood flow reversal from the aorta into the left ventricle (LV), AI leads to progressive LV dilation and eventual heart failure if left untreated. Frequently encountered with TAA involving the aortic root [5, 6, 7], AI is a relatively common condition with a 13% male and 8.5% female prevalence [8]. In contrast, AS pathophysiology resembles atherosclerotic disease (lipid accumulation, inflammation, fibrosis, and calcification), where leaflets progressively stiffen, reducing blood efflux, causing pressure overload and LV myocardial hypertrophy [9, 10]. Affecting 3–5% of people >65 years of age, AS severity and prevalence increases with age [11]. Compared to normal or sclerotic AV (early stage AS), AS is associated with an increased incidence of dilated ascending aortas [12].

**Fig. 1.**
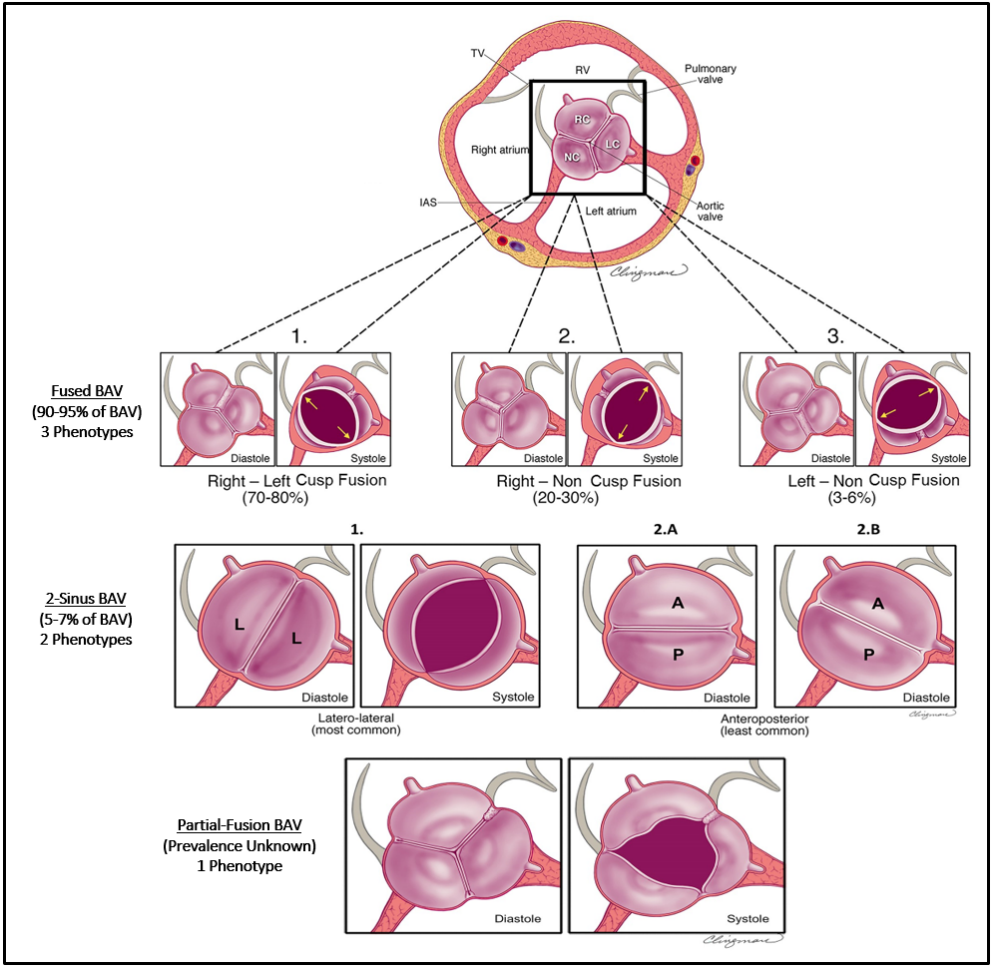
**Schematic of the three major BAV types with associated phenotypes**. BAV types as seen by short-axis transthoracic echocardiogram. (Top Row) Fused BAV type is the most common with 3 phenotypes named according to cusp fusion pattern. A raphe may not always be visible or present, however all fused BAV have 3 distinguishable aortic sinuses, with the non-fused cusp typically being the largest. (Middle Row) 2-sinus BAV is uncommon, does not have a raphe, and is characterized by 2 cusps of nearly equal size and shape, each occupying 180° of the circumference and has only 2 distinguishable aortic sinuses. Relative cusp orientation dictates phenotype as either latero-lateral or anteroposterior. Coronary arteries arise from each cusp (1 and 2A) or both from the anterior cusp in the AP phenotype (2B). (Bottom Row) Partial-fusion BAV (or forme fruste) is characterized by the presence of a short cusp fusion (<50%) at the base of a commissure in an otherwise normal appearing tricuspid aortic valve with 3 symmetrical cusps. Abbreviations: A, anterior; BAV, bicuspid aortic valve; IAS, interatrial septum; L, latero-lateral; LC, left coronary cusp; NC, non-coronary cusp; P, posterior; RC, right coronary cusp; RV, right ventricle; TV, tricuspid valve. Reproduced and modified with permission from the authors [15].

BAV occur in 1–2% of the population, carry a 3:1 male predominance [13], and are the most common congenital heart defect [14]. Until recently, there has been no consensus on the nomenclature and classification of different BAV types, with numerous heterogeneous classification systems causing confusion [15, 16]. With international consensus, congenital BAV are now classified into one of 3 major types (Fused BAV, 2-Sinus BAV, and Partial-fusion BAV), each with specific phenotypes (Fig. 1, Ref. [15]). As a congenital condition with strong genetic ties, BAV are associated with manifestations in tissues beyond the AV, including: aortopathies, aortic valvulopathies (AS and/or AI), additional congenital cardiovascular abnormalities, coronary anomalies, and other genetic disorders [17, 18]. Specifically, mutations associated with BAV development also impact aortic architecture, increasing susceptibility to TAA formation and dissections, while altered hemodynamics across bicuspid shaped AV further contribute to aortic dilatation. AV disease also develops much earlier in BAV, with AS occurring most frequently (>70%), followed by AI (15–30%), and mixed AI/AS (20%) [15, 18].

Given the asymptomatic nature of TAA, serial surveillance after diagnosis using various imaging techniques like echocardiography, computed tomography (CT), and magnetic resonance imaging (MRI) is crucial. However, accurately predicting disease progression and the risks of AAE in TAA patients, especially when there is concurrent AV disease, remains exceedingly challenging. Currently, there is no comprehensive approach in managing patients with AV disease and TAA that incorporates all imaging techniques and necessary knowledge concerning AV disease-TAA pathophysiology; an approach essential to providing accurate disease prognosis and appropriate monitoring in these patients.

This review aims to summarize the latest scientific knowledge on the link between AV disease (AI, AS, BAV) and aortopathies of the proximal aorta (root/ascending), as well as identifying current gaps in the management of TAA patients with AV disease. We hope the manuscript will set the stage for further research to better address these complex conditions that existing clinical tools and methodologies fail to do.

## 2. Connecting Aortic Valve Pathology with Thoracic Aortic Aneurysm

The most proximal portion of the aorta is known as the aortic root, starting with the anatomical crown-shaped annulus of the AV cusp insertion points or virtual basal ring, followed by the ventriculoaortic junction, the AV leaflets housed within the sinus of Valsalva, and ending with the sinotubular junction (STJ). From there, the ascending tubular aorta begins and courses until the aortic arch, defined as the takeoff of the innominate artery. Normal mean aortic root diameters range from 3.50 to 3.91 cm (smaller in women) and taper in the ascending aorta to 2.7 and 3.0 ± 0.4 cm in women and men with tricuspid aortic valves (TAV) respectively. By convention, an arterial aneurysm is defined as any artery dilated to at least 1.5× its expected normal diameter [19], and although this definition works for aneurysms of the descending and abdominal aorta, we now know it fails when defining aneurysms of the root and ascending aorta [20].

When determining if an aortic root or ascending aorta is aneurysmal, the most important consideration to account for is the natural history of abnormal aortas in these locations, specifically the relationship between aortic diameters (+/– presence of BAV) and the incidence of adverse aortic events, as guideline recommendations for surgical intervention are based on this. By evaluating the risk of type A dissections below the traditional 5.5 cm threshold for prophylactic aortic repair, Paruchuri *et al*. [21] found that when compared to control aortic diameters of <3.4 cm, aortic diameters between 4 and 4.4 cm conferred an 89-fold increase in relative risk of dissection, and those ≥4.5 cm carried a 6000-fold increase. Consequently, the most recent 2022 American College of Cardiology (ACC)/American Heart Association (AHA) Guidelines for the Diagnosis and Management of Aortic disease now define dilatation of the root or ascending aorta as diameters between 4.0–4.4 cm and aneurysms as those with diameters ≥4.5 cm [20]. This definition is also now consistent with that proposed by the 2014 European Society of Cardiology aortic disease guidelines [22].

For patients whose height and weight are significantly different from the average population (≥1–2 standard deviations ± mean), it is important to normalize aortic diameters in order to accurately differentiate between normal and dilated/aneurysmal aortas. Various normalization methods exist, including aortic size index (ASI) and height index (AHI), where the ratio of aortic diameter to body surface area (ASI) or height (AHI) is calculated [23, 24]. Another commonly used method utilizes the cross-sectional area (CSA) of the aorta, rather than aortic diameter, to normalize aortic size to height [25]. These measures are frequently used in clinical practice for adult patients with TAA, as they have been shown to be more reliable predictors of AAE than diameter alone [21, 22, 23]. Consequently, the most recent ACC/AHA guidelines recommend using indexed aortic measures, including ASI ≥3.08 cm/$m^{2}$, AHI ≥3.21 cm/m, and CSA to height ratio ≥10 ${\mathrm{cm}}^{2}$/m, as new thresholds for surgical intervention [20].

The formation and particular location of an aneurysm can both influence and be influenced by AV morphology and pathology. In AS, altered blood flow through a stenotic valve leads to a forceful ejection jet, altered hemodynamics, and mechanical stresses on the aortic wall distal to the stenosis. This is ultimately associated with proximal aortic dilation and aneurysm formation, in a concept known as post-stenotic dilation [26, 27, 28]. The extent of this relationship is even more apparent in patients with BAV and AS, so much so, that this phenomenon is defined as BAV–associated aortopathy. BAV–associated aortopathy most commonly affects the tubular ascending aorta, occurring in up 60–70% of BAV patients [29, 30], and is greatest with right-left (RL) coronary cusps are fused, followed by right-non (RN) coronary cusp fusion [31, 32]. Interestingly, within the BAV population, aortic dilation is present in 40% of patients regardless of the presence of AI/AS, raising the possibility of genetic or pathological changes related to the development of BAV that also lead to aortic wall weakness and aneurysm formation [29, 33, 34]. The relative contribution of hemodynamic forces and genetics to the development of BAV-associated aortopathy remains debated [29, 35], with both factors likely contributory.

**Fig. 2.**
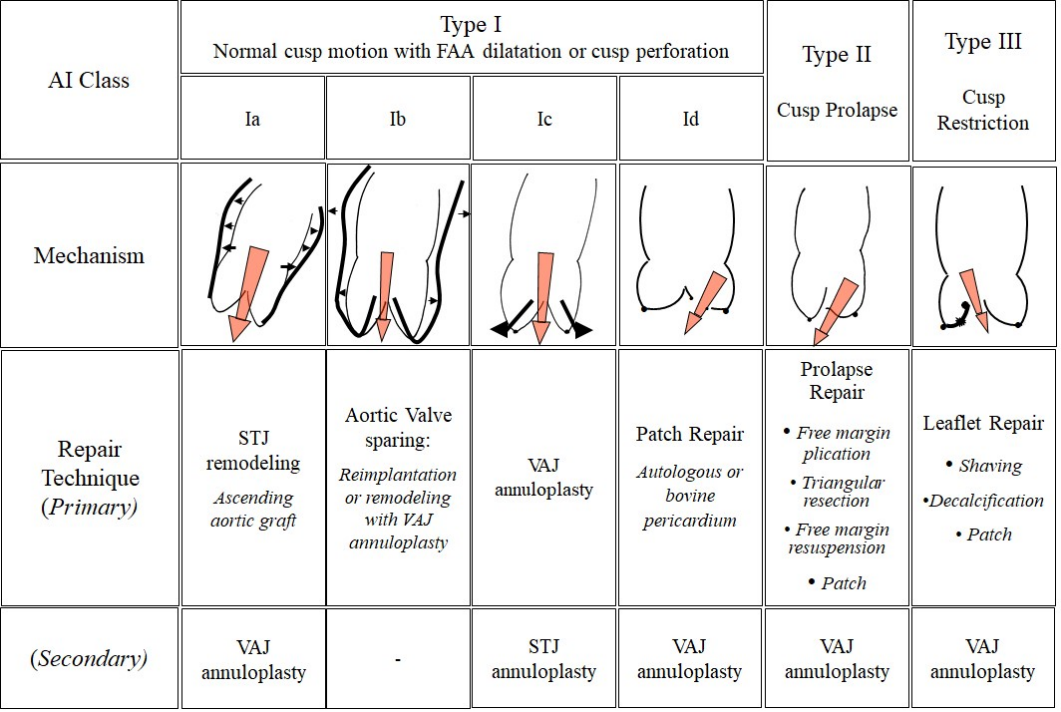
**Repair-oriented functional classification of AI with disease mechanism and repair techniques**. Abbreviations: AI, aortic insufficiency; FAA, functional aortic annulus; STJ, sinotubular junction; VAJ, ventriculoaortic junction. Reproduced and modified with permission from the authors [36].

Conversely, aneurysms involving the STJ, sinuses of Valsalva, and/or aortic annulus often result in the development of AI (Type 1a-c), where the AV leaflets are pulled apart and no longer able to coapt (Fig. 2, Ref. [36]). Since AI is associated with aortic dilation, a vicious cycle of worsening AI and aneurysmal degeneration can ensue. With progressive dilatation of the aortic root, the AV leaflets become stretched and irreversibly damaged, leading to leaflet fenestrations, cusp prolapse [36, 37, 38, 39], and worsening AI.

## 3. Clinical Patterns of TAA Depend on Valvular Dysfunction

**Fig. 3.**
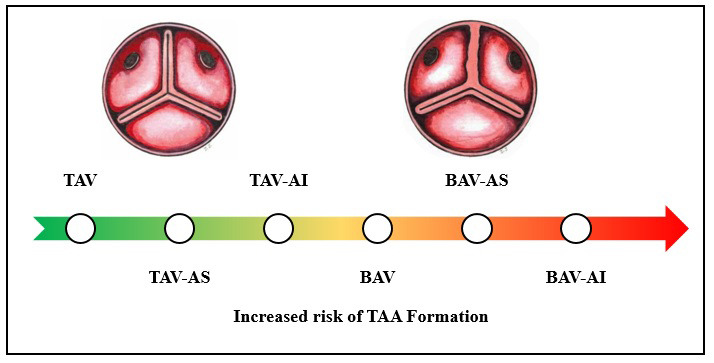
**Proposed Spectrum of TAA formation risk in the presence of AV disease**. Abbreviations: AV, aortic valve; AI, aortic insufficiency; AS, aortic stenosis; BAV, bicuspid aortic valve; TAA, thoracic aortic aneurysm; TAV, tricuspid aortic valve.

The natural history and risk profile of an aneurysm change drastically whether associated with TAV or BAV, as well as the presence of AS or AI. On one end of this spectrum, TAV-AS aneurysms tend to be slow-growing with more stable aortic walls, whereas, on the other extreme, BAV-AI aneurysms are particularly aggressive (Fig. 3). Between these, less is known about the effects of TAV-AI and BAV-AS on TAA development, and while not as dangerous as BAV-AI, both are prevalent and remain dangerous [27, 37, 38, 39, 40, 41, 42].

### 3.1 Tricuspid Aortic Valves and TAA

Several key clinical studies have examined the impact of AS/AI in BAV/TAV on aortic aneurysm formation and progression. The aortic wall of TAV-AS patients remains relatively stable in contrast to those with BAV-AS, with aortic dilation occurring at slower rates in TAV-AS patients [40, 41]. After undergoing aortic valve replacement (AVR) for severe TAV-AS in patients without aortic aneurysms, aortic growth rates were found to be significantly slower at 0.09 mm/yr, whereas BAV-AS patients demonstrated progressive aortic dilation of up to 0.36 mm/yr (*p* < 0.001) [41]. Additional studies have further suggested a protective effect to AVR on aortic dilation when performed in patients with TAV-AS, with patients demonstrating no further aortic dilation post AVR [40]. This however was not demonstrated in BAV-AS patients, with BAV patients showing similar progressive dilation irrespective of AVR.

The impact of AI in TAV patients on the development of TAA or risk of AAE remains to be thoroughly explored. A recent small study (n = 36) by Balint *et al*. [43] examining this relationship demonstrated that the presence of AI in TAV patients was significantly associated with medial degeneration of the ascending aortic wall (even in the presence of normal-sized aortas), when compared to TAV patients without AI. Using histological and immunohistochemical analyses, the authors further demonstrated more pathological aortic remodeling in TAV-AI patients compared to TAV-AS patients, including: increased mucoid extracellular matrix accumulation, elastin loss and fragmentation, and decreased fibrillin and collagen expression. As such, TAV-AI patients appear to be at increased risk of TAA formation compared to both TAV and TAV-AS patients, which is consistent with what is observed in patients with BAV and AI vs AS [37, 44, 45].

### 3.2 Bicuspid Aortic Valves and TAA

Unlike TAV disease, aneurysms associated with AI vs AS in patients with BAV have been well studied. With a higher prevalence of aortic dilatation, more severe pathological aortic remodeling, and a higher probability of adverse aortic events, BAV-AI patients possess the worst clinical course compared to BAV-AS and functionally normal BAV [37, 39, 42, 44, 45]. This is due to a combination of (i) increased hemodynamic burden secondary to the increased stroke volumes in AI, and (ii) intrinsic abnormalities found in the aortic walls of BAV patients leading to fragility [27]. Patients with BAV-AI are more often male and younger than BAV-AS [37, 46], and usually associated with root dilation (root phenotype) compared to predominantly tubular ascending aortic dilation in BAV-AS patients [38, 47, 48]. Echocardiography data from the early 1990s showed BAV-AI was associated with a higher prevalence of aortic annular (59% vs 9%) and sinuses of Valsalva dilatation (78% vs 36%) when compared to BAV-AS, while 60–65% of both groups had ascending aortic dilation [48].

Similarly, Sievers *et al*. [38] also demonstrated associations of BAV-AI with root/ascending dilation and BAV-AS with eccentric ascending aortic dilation. Notably, even BAV patients with only trace AI were still significantly associated with root/ascending aortic dilatation, emphasizing the more aggressive aortopathy phenotype found in BAV-AI [38]. Expanding on this, Della Corte *et al*. [47] poignantly showed BAV-AI to be predictive of root dilation (odds ratio (OR) 3.9), while BAV-AS was predictive of mid-ascending aortic dilation (OR 23.8) and protective of root dilation (OR 0.26). Furthermore, the frequency of aortic replacement at time of BAV surgery is significantly higher with BAV-AI patients when compared to BAV-AS patients (35% vs 17%, (*p* < 0.001) [37, 38].

**Fig. 4.**
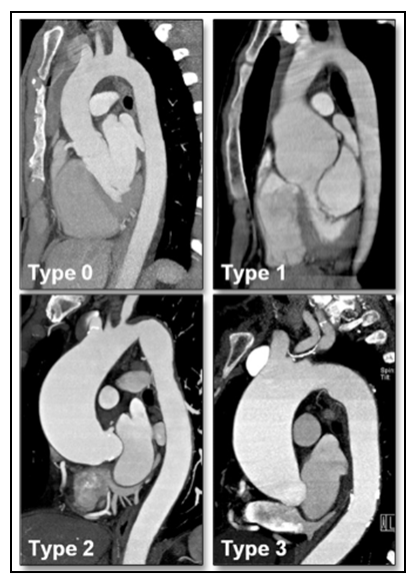
**MDCT images representative of BAV aortopathy phenotypes**. Bicuspid aortopathy phenotype is dependent on the pattern of BAV dysfunction, including both anatomical BAV configuration and the presence of AI or AS. Three distinct phenotypes have been identified, including: Type 0—normal aorta, Type 1—dilated aortic root only, Type 2—involvement of the tubular portion of the ascending aorta, and Type 3—diffuse involvement of the entire ascending aorta and transverse aortic arch. Reproduced and modified with permission from the authors [49]. Abbreviations: AI, aortic insufficiency; AS, aortic stenosis; BAV, bicuspid aortic valve; MDCT, multi-detector computed tomography.

Interestingly, the configuration of BAV cusp fusion has also been shown to influence resultant valve dysfunction type (AI vs AS) and aortopathy phenotype. Using the Sievers classification system for BAV phenotype, Sievers *et al*. [38] demonstrated stenotic BAV (type 0 and type 1 RL) to be significantly associated with more localized aortic dilatation (ascending only), whereas insufficient BAV type 1 RL tended to involve the root and showed more extended aortopathy (root and ascending aorta). Categorizing BAV type based on orientation of the free edge of the cusp, Kang *et al*. [49] found AI significantly more prevalent in anterior-posterior vs RL configuration (anteroposterior (AP) 32.3% vs RL 6.8%, *p* < 0.0001), while AS was more common in RL vs AP (66.2% vs 46.2%, *p* = 0.01). Comparing aortopathies, these authors found BAV-AP was more common in normal aortas or aortic root dilation (type 0/1 aortopathy), and BAV-RL with ascending or ascending/arch dilation (type 2/3 aortopathy) (Fig. 4, Ref. [49]). Completing this interconnected triangle, AI was significantly more common in type 0/1 aortopathy (32.9% vs 10.2%, *p* < 0.0001), and AS with aortopathy type 2/3 (64.8% vs 44.3%, *p* = 0.002) [49]. Since, RL fusion as defined by Sievers (type 1 RL) was included in Kang *et al*.’s [49] AP group, and RN (Sievers type 1 RN) was part of their RL group, both studies correlate well and show a strong clinical connection between BAV cusp configuration, valvular pathology, and aortopathy phenotype.

Current criteria for concomitant aortic replacement when undergoing surgery for AV dysfunction is 4.5 cm, irrespective of AV anatomy or dysfunction type, holding a Class 2a recommendation for both TAV and BAV [20]. As such, this recommendation fails to account for the increased risks of TAA formation and adverse aortic events seen with BAV patients, as well as type of valve dysfunction present. This recommendation was largely based on a small study of 200 patients by Borger *et al*. [50], where they demonstrated a significantly increased risk of aneurysm, dissection, or sudden death (*p* < 0.001) in BAV patients with aortic diameters between 4.5 to 4.9 cm, compared to those with aortas <4.5 cm at 15 years following AVR. However, this study did not assess the associations of AI or AS on these outcomes.

With the same 4.5 cm recommendation for prophylactic aortic replacement as TAV, a significant cohort of already at risk BAV patients with dilated aortas are left behind, who may be at even higher risk depending on the presence of BAV-AI. Comparing BAV-AI to BAV-AS patients post-surgical AVR, BAV-AI patients showed faster rates of aortic dilation (0.29 mm/yr vs 0.18 mm/yr, *p* < 0.001) and increased occurrence of adverse aortic events (15.5% vs 4.5%, *p* = 0.018) [39]. BAV-AI is an independent predictor for adverse aortic events even after AVR, with patients showing a 10-fold higher risk of dissection than BAV-AS patients post AVR (2.8% pooled estimate of dissection rate vs 0.2%), with increasing risk seen with smaller aortic diameters in BAV-AI patients [42]. Despite these findings, both groups demonstrated similar long-term survival [51], likely due to the overall low numbers of observed adverse aortic events.

## 4. Hemodynamic Changes in the Ascending Aorta in the Setting of AS/AI, TAV/BAV, and Impact on the Aortic Wall Remodeling

Altered blood flow through aneurysmal aortas cause hemodynamic changes that affect the aorta, even in the absence of AV disease (AS, AI, or AS/AI) or abnormal AV morphology (BAV). With the advent of 4D MRI, a great deal of research in fluid dynamics has been produced, as blood flow through the heart and great vessels over an entire cardiac cycle can now be evaluated [52]. As expected, 4D MRI of TAV-TAA patients has demonstrated wall shear stress (WSS) reduced by 21% to 33% across most regions of dilated aortic walls relative to non-dilated aortas [53]. Holding stroke volume constant, mean velocity gradients are reduced in the presence of an enlarged vessel, which in turn reduces WSS [54]. The reduced pressure gradient is secondary to aberrant flow within the dilated aorta, where the incidence and strength of supraphysiologic helix and vortex flow correlates with increased ascending aortic diameter [55]. Moreover, systolic time to peak velocity and extent of retrograde flow both increase with increasing aortic diameter, leading to reduced flow efficiency in TAA [56].

Several studies have demonstrated altered flow dynamics in AS to impact the aortic wall [53, 57]. Bauer *et al*. [57] compared patients with BAV-AS to those with TAV-AS, and demonstrated no differences in aortic root diameter between groups, however the peak systolic wall velocity in the anterolateral region of the aortic wall was higher in BAV-AS than TAV-AS [36]. Within BAV-AS, velocity was higher in anterolateral than the posterolateral location [57]. However, these authors did not have a BAV group with no stenosis, so it remains unclear whether this difference was due to BAV phenotype alone. To isolate these confounding factors, van Ooij *et al*. [53] analyzed BAV and TAV patients with and without AS. In mild stenosis, TAV patients with TAA go from decreased WSS to increased WSS along the outer portion of the ascending aorta. As stenosis progresses to moderate or severe, impaired valve opening leads to more pronounced high velocity jets with marked increase in regional WSS.

Remarkably, differences in WSS location between BAV and TAV dissipated when the degree of AS was moderate/severe, implying AS as the now dominant factor governing hemodynamics, as well as it being a contributing factor in TAA formation [53]. How this altered flow affects aortic growth over time would require longitudinal imaging studies, which have yet to be performed. In addition, flow dynamic studies assessing TAA formation in the presence of AI are lacking in both TAV and BAV patients [58].

Aside from genetic components implicated in the development of BAV-associated aortopathy, altered hemodynamics play a large role in TAA formation in both TAV and BAV patients. These effects are more pronounced in BAV patients and also vary depending on the presence of AI or AS. In contrast to TAV, where a central flow jet directs blood flow parallel to the aortic wall, BAV usually produce eccentric outflow jets [53, 59, 60, 61] which is consistent with the asymmetric aneurysmal formations characteristic of BAV [62]. Compared to TAV, averaged WSS is elevated in BAV irrespective of aneurysmal formation or valvular pathology [59, 63]. Flow displacement (eccentric jets) is higher in BAV and is predictive of aortic growth rate, with dilation rates up to 1.2 mm/yr in patients with markedly eccentric flows relative to 0.3 mm/yr in BAV patients with les flow displacement [64, 65]. BAV have decreased cusp opening angles (a measure for BAV opening restriction), which causes systolic flow deflection toward the right anterolateral ascending wall [66]. This measure also independently predicts ascending diameter and growth rate in non-dilated aortas.

**Fig. 5.**
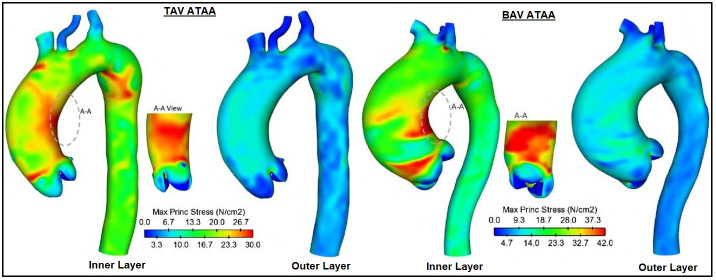
**Computational FSI analysis for inner and outer maximum WPS in ATAA patients with TAV and BAV**. Both TAV and BAV patients demonstrate higher inner WPS compared to the outer aortic wall, with local maxima of WPS occurring just above the STJ (inset image). BAV patients display slightly higher stresses than TAV patients (36.5 N/${\mathrm{cm}}^{2}$ vs 29.4 N/${\mathrm{cm}}^{2}$), suggesting a greater risk of aortic dissection. Reproduced and modified with permission from the authors [61]. Abbreviations: ATAA, ascending thoracic aortic aneurysm; BAV, bicuspid aortic valve; FSI, fluid structure interaction; N/${\mathrm{cm}}^{2}$, newton per centimeter squared; STJ, sinotubular junction; TAV, tricuspid aortic valve; WPS, wall principal stress.

Like wall shear stress, the concept of wall principal stress (WPS) is an important factor in understanding the mechanical behavior of TAA, and also differs between BAV and TAV. In contrast to WSS, WPS denotes the location of maximum aortic wall shear stress, and is perpendicular to the direction of blood flow rather than parallel [58, 61, 67]. Irrespective of AV type, WPS is greater along the inner aortic wall when compared to the outer wall, with local WPS maxima occurring just above the STJ (Fig. 5, Ref. [61]) [68]. It is at this location that an aortic wall is mostly likely to tear or rupture, secondary to the discontinuities in stress at the interface between aortic layers [61]. This is supported by clinical observations noting this location as the most common origin site of type A dissections [61, 69]. Lastly, with respect to valve type, BAV aneurysms exhibit higher severity WPS at all locations when compared to TAV [61], which may account for the increased risks of dissection among patients with BAV [33, 70].

Further complicating the hemodynamic role in BAV is the recognition that cusp fusion phenotype changes the outflow jet orientation and flow abnormalities, impacting the aorta and the WSS parameters [53, 60, 71]. The two most common cusp fusion types found in BAV is RL fusion, followed by RN coronary cusp fusion. Blood flow through BAV-RL occurs as right-handed helical flow, with right-anterior flow jets, whereas right-non-coronary (R-NC) has more severe flow abnormalities, and gives rise to a left helical flow and left-posterior or right-posterior flow jet [60, 71, 72]. These differences lead to different areas of aortic WSS. BAV-RL aortas have peak WSS along the right-anterior ascending aorta [59, 60], or increased WSS at the root and along the entire outer curvature of the aorta [53]. In contrast, BAV-RN leads to peak WSS along the right-posterior aorta [60], or increased WSS at the distal portion of ascending aorta [53]. These differences correlate well with clinical presentations associated with cusp fusion phenotype, namely RL fusion being associated with a root dilation phenotype, and RN with distal ascending aorta dilation and often root sparing [53].

Flow alterations are more pronounced, and different from each other, when assessing the combined effect of BAV and the presence of AS or AI. Shan *et al*. [59] observed that compared to control BAV, BAV-AI patients had universally elevated WSS and correlated with stroke volume. BAV-AS patients had elevated flow eccentricity, as the accelerated flow velocity from the AS exacerbated the already eccentric flow found with BAV. However, the location of peak WSS at the right-anterior ascending aorta, was similar regardless of AI or AS, as was the associated aortopathy, mainly type 2. Since this study focused solely on BAV R-L patients, the location of peak WSS was likely due to this phenotype rather than AI or AS [59]. As such, further studies correlating the effects of valve dysfunction type (BAV-AI and/or AS) on altered hemodynamics and not just cusp fusion phenotype are needed. In addition, longitudinal imaging studies comparing the impact of AI and AS flow dynamic on the aortic wall are needed to help explain the observed differences in natural histories of aortopathies in the presence of AI vs AS.

## 5. Understanding the Impact of Aortic Valve Morphology and Function on the Integrity of the Ascending Aorta

With an abundance of evidence, it is clear that AV structure and function greatly influences the integrity of the aorta. The association between AS and TAA, as well as AI and TAA in the setting of TAV or BAV has been thoroughly confirmed. However, the exact mechanisms through which each valvular anomaly contributes to aortic dilation and aneurysm formation remain unclear. While examinations of AV and aortic anatomy, have revealed similarities in cellular and extracellular matrix compositions, the extent to which TAA pathogenesis in the setting of AS/AI is caused by genetic alterations (heritable gene mutations causing aortic wall fragility), or altered hemodynamics (WSS), or both, continues to be a debate.

### 5.1 Aortic Valve and Aortic Embryology and Anatomy

The AV arises from the semilunar cushions, structures that form early on during embryonic heart development. These cushions consist primarily of myocytes (neural crest origin, secondary-heart field origin), endocardial/endothelial cells, and a hyaluronic acid-rich matrix. Through cell proliferation, differentiation, and matrix remodeling, the semilunar cushions give rise to the mature AV, which consists of three layers. The fibrosa layer is located on the ventricular side of the AV and is rich in collagen providing tensile strength and flexibility. The middle layer, or spongiosa, contains less collagen with a high abundance of proteoglycans and water retention, creating a more compressible matrix to the AV. Lastly, the ventricularis layer is adjacent to blood flow in the aorta and largely composed of elastin providing flexibility to the AV leaflets [73].

In contrast, development of the proximal aorta begins as a single tract outflow structure arising from the right and left ventricles, eventually dividing into two separate vascular channels (aorta and main pulmonary artery) with the formation of the aorticopulmonary septum [74]. Once fully developed, the ascending aorta also contains three main layers: (i) the innermost layer is known as the tunica intima and is in direct contact with blood. Made up of a single layer of endothelial cells this is also the weakest layer, (ii) the tunica media makes up the middle layer of the aorta and contains >50 layers of alternating smooth muscle cells, elastic fibers, and collagen type I/III, providing strength and distensibility to the aortic wall, lastly (iii) the outermost layer or tunica adventitia is made of a thin layer of collagen, houses the vasa vasorum, and considered the strongest layer of the aorta, possessing the greatest tensile strength.

### 5.2 Fluid Shear Stress in Vasculature

Aforementioned, although the exact mechanisms (and contributions of each) underlying aortic aneurysm formation have yet to be fully elucidated, the concept of fluid shear stress has been implicated as another important contributing factor, and links both aortic valve and aortic wall pathological changes [58, 67, 75, 76]. Both fluid and WSS are two related, but distinct concepts in the field of cardiovascular physiology and biomechanics. While WSS refers to the force exerted on the inner wall of a blood vessel by the fluid flowing through it, fluid shear stress results from friction between the fluid and the surface of the blood vessel, and plays an important role in maintaining normal healthy vascular biology and cardiovascular physiology [58, 67, 76].

As a consequence of similar anatomy, the endothelial linings and extracellular matrix components of both the aortic valve and aorta are affected by fluid shear stress. While the effects of fluid shear stress (FSS) at the cellular level on these components and their role in exacerbating disease progression are still being researched, it is widely recognized that the physical forces produced by fluid shear stress play a significant role in the development and progression of aortic aneurysm formation [75, 77]. Furthermore, fluid shear stress may also lead to changes in the mechanical stress on aortic valve tissue, potentially resulting in pathological changes, such as valve stenosis or regurgitation, as well as structural valve degeneration [75, 78]. Lastly, the location and magnitude of these forces depend on factors such as pre-existing aortic aneurysms, the presence of AS or AI, and the morphology of aortic valve, specifically BAV [60, 79].

### 5.3 What We Know So Far?

To date, most human studies evaluating the effect of AV disease on the ascending aorta have only been descriptive histological studies, with no mechanistic interrogations on the pathogenesis of AV dysfunction causing aortopathies. While animal models to study TAA and valvular pathologies exist, they are limited and unable to replicate all the different phenotypes observed clinically.

Miura *et al*. [80] compared AV with AS and AI in elderly patients, using scanning acoustic microscopy and immunohistochemistry analysis. AS valves presented thick nodular leaflets with active fibrosis and calcification, and a stiff fibrosa layer lacking collagen I but rich in collagen III. AI valves were thin but stiffer, contained collagen type I and III in the fibrosa, as well as progressive accumulation of advanced glycation end-products, which are non-enzymatic modifications of proteins [81] that strongly contributes to structural and functional degeneration in various native tissues and diseases [82] and contribute to stiffness [83, 84].

Given the incidence of ascending aortopathies increases in the presence of valve anomalies, it would seem logical to evaluate the AV and the ascending aorta as one single entity. Aforementioned, Balint *et al*. [43] previously demonstrated an increased risk of ascending aortic dilation and rupture in TAV patients with AI and non-dilated aortas using this methodology. These results were further confirmed in a larger, more recent study by Sequeira Gross *et al*. [85] that examined the association of valve dysfunction (AI vs AS) and valve morphology (TAV vs BAV) on aortic remodeling in 131 patients referred for AVR. Results from this study uncovered an increased expression of all medial degeneration and inflammatory markers in the aortas of the AI group when compared to AS-aortas. Patients with BAV-AI were significantly younger than those with BAV-AS, but not microstructural differences were noted between BAV-AS and BAV-AI. Within the AI group, markers for medial degeneration, were increased in TAV-AI versus BAV-AI [85]. The clinical ramifications of these findings remain unknown.

Whether the presence/type of valvular abnormality has a direct effect on TAA formation/progression or not, and whether or not interventions on TAA should be undertaken when present or depending on type of AV dysfunction, during AV surgery remains highly debatable. A study to examine this by Roberts *et al*. [45], evaluated the relationship between AV structure and excised portions of aneurysmal ascending aorta in surgical patients with AS (±AI) vs patients with pure AI. The AV was congenitally malformed in 98% of AS patients (unicuspid or bicuspid), and 60% of AI patients (bicuspid). Unadjusted analysis of these patients showed a significantly higher likelihood of ascending aortic medial elastic fiber loss (EFL) in AI patients when compared to AS and control valves, strongly suggestive that type of AV dysfunction may aid in predicting loss of aortic medial EFL in patients with AV disease and concomitant TAA [45]. EFL has also been assessed in the setting of BAV, comparing patients with AS and AI undergoing AVR and simultaneous replacement of the proximal aorta for aortic diameters ≥50 mm [44]. Results of this study also demonstrated higher rates of moderate/severe aortic EFL was associated with BAV-AI when compared to the BAV-AS [44].

## 6. Future Research Perspective

Despite remarkable progress in the past few years in the understanding of the pathophysiology of TAA, the exact causes and pathways underlying the phenotypic differences observed in AS/AI and TAV/BAV TAA patients remain undefined. This is likely due to the multifactorial nature of such diseases, where genetic and hemodynamic factors together dictate the fate of disease progression.

Lineage tracing analyses using reporter genes, and studies of conditional knockout animal models have revealed the presence of common cellular origins contributing to the formation of both the ascending aorta and the leaflets of the AV (smooth muscle cells derived from the secondary heart field and cardiac neural crest cells) [86, 87, 88]. Whether this common cellular origin plays a contributing role in the pathophysiology of TAA remains to be answered.

Endothelial cells represent the interface between blood and the aortic wall and valve. As such, these cells are the first to be exposed to shear stress generated by blood flow. Changes in shear stress can lead to changes in endothelial cell gene expression and function, with different responses observed when laminar flow versus oscillatory flow have been tested on these cells [89, 90]. Interestingly, laminar shear stress induced differential responses in porcine endothelial cells derived from the aortic wall to those derived from the AV [91] and transcriptional differences have been highlighted between these two cellular populations [92]. More research focusing on understanding human endothelial cells and smooth muscle cells derived from the aorta and the AV, as well as the implications of BAV genetic background, should be undertaken to help explain the clinical variability that we see on imaging. This knowledge will help bridge the gap and integrate our clinical understanding with the findings from basic science which may help in the management of patients with TAA and AV disease.

### Genetics of BAV and Associated Aortopathy

Human and genetic studies continue to shed new light on the molecular pathogenesis and development of BAV. Primarily inherited as an autosomal dominant trait, BAV inheritance displays incomplete penetrance and variable expressivity due to the complex genetic architecture of its numerous interacting genes [93, 94]. As such, BAV may also arise in other genetic syndromes, particularly Turners syndrome [95] and connective tissue disorders (Loeys-Dietz, Marfan, vascular Ehlers-Danlos) [94, 96, 97], all of which are already linked to TAA formation [98].

As outlined in this review, the presence of a BAV is associated with serious long-term health risks including progressive aortic valve disease and thoracic aortopathy, with approximately 30–40% of BAV patients undergoing TAA repair [14, 99]. When compared to TAV patients, BAV patients (with or without aneurysms) are at increased risk of future aortic dilation and dissection [33, 70], and display faster rates of aneurysmal growth [20, 51]. These associations are so strong that, even after aortic valve replacement, BAV patients still require lifelong surveillance of the aorta [20, 51].

Given the significant genetic associations of BAV, and the potential lethality of BAV-associated aortopathy complications (dissection/rupture), current guidelines recommend screening of all first-degree relatives with transesophageal echocardiogram (TEE) for the presence of a BAV and/or proximal aortic dilatation for BAV patients with associated aortopathy (Class I) and without (Class IIa) [20, 99]. In contrast, no established protocols for providing genetic counseling to individuals and families affected by BAV exist. This is a result of the current poor understanding of BAV genetic etiology [100, 101], which is further complicated by a complex coexistent genetic association with diseases of the aorta and cardiac development [100, 102]. As such, intense work on the genetic origins underlying the pathogenesis of BAV-associated aortopathy is currently ongoing [101], in the hope that genetic risk factors may be identified for use in screening tools to not only help identify BAV patients at risk of complications but also in family member prevention.

Multiple human chromosomal regions (18q, 5q, 13q [103]) and gene mutations (*GATA5 * [104, 105] and *MATR3 * [104]) have been identified in the pathogenesis of BAV, with the most well-described being the *NOTCH1* gene. *NOTCH1* codes for a transmembrane receptor involved in organogenesis [106], promoting endothelial to mesenchymal transition, and plays a critical role in cardiac valve development and valve calcification [101, 106]. Mutations in *NOTCH1* pathway related genes contribute to left ventricular outflow tract (LVOT) obstructive phenotypes such as BAV development [93] and accelerated calcium deposition of the aortic valve [106]. *NOTCH1* is also associated with non-syndromic BAV in a limited number of familial cases and ~4% of sporadic cases [14, 105].

Mutations in transforming growth factor-β signaling pathway, such as transforming growth factor-beta (TGFB) 2 ligand and receptor that cause Loeys-Dietz syndrome (*TGFBR1*, *TGFBR2*, *TGFB2*, *TGFB3*) have also been shown to have a higher prevalence of BAV (4–15%) [15, 107]. *ACTA2* and *SMAD6* mutations, which cause heritable thoracic aortic aneurysms and dissections, have also been identified in non-syndromic BAV (*SMAD4* and *SMAD6*) and TAA (*ACTA2*) [93]. *Fibrillin1* (*FBN1*) mutations, responsible for the development of Marfan syndrome, have also been found to be associated with BAV development independent of Marfan [97]. Aneurysm formation in BAV patients has also been linked to patients with polymorphisms in *eNOS*, *angiotensin-converting enzyme (ACE)*, *matrix metalloproteinase (MMP) 9* and *MMP2 * [103, 108].

While current evidence supports the involvement of a genetic basis in the pathogenesis of BAV-associated aortopathy [61, 94, 101], due to complex heterogeneity, multiple signal pathway involvement, and numerous mutations in diverse genes [101], causative genes remain largely unknown in most cases. Consequently, molecular testing in BAV currently remains low yield. Although some argue genetic screening can lead to reduced healthcare costs, by eliminating surveillance imaging negative patients [93], this has not been validated and may have harmful consequences. For instance, patients with BAV and a gene that was not tested for could be wrongly denied care. Furthermore, transthoracic echocardiogram (TTE) screening of first-degree relatives of BAV patients to detect BAV and aortopathy has already been demonstrated to be cost-effective [109]. While genetic testing sounds promising, until new BAV causing genes are discovered, specifically those linked to the development of AV disease and/or aortopathy, genetic testing should be reserved for BAV patients with features of genetic syndromes or heritable TAD [93], and not used in family screening.

## 7. Discussion/Conclusions

Current guidelines for aortic replacement in TAA do not account for the presence or type of AV dysfunction when determining aortic size thresholds for surgery [20, 110], and vice versa, with AV disease guidelines providing no recommendations for aortic interventions during AV surgery depending on valve dysfunction type [111]. Specifically, prophylactic repair of TAA is recommended at ≥4.5 cm if undergoing AV surgery, irrespective of whether the valve is bicuspid or tricuspid, regurgitant or stenotic [20, 112]. Developing a framework to understand the impact of valvular dysfunction on TAA formation, with clinical implications on surveillance, both before and after surgery, and need for surgery itself, is critical. This review clearly demonstrates epidemiological and clinical phenotypes connecting AI in both TAV and BAV with major adverse aortic events, as well as more rapid rates of TAA growth. Patients with AI are at increased risks of developing aortopathy at younger ages, increased risks of root dilation, rapid rates of TAA growth—both before and after AVR, and carry a greater risk of adverse aortic events (Tables 1,2). These risks are further exacerbated in patients with BAV-AI compared to TAV-AI. Furthermore, in addition to the already increased hemodynamic burden from AI on the aortic walls, AI patients have universally elevated WSS and more severe medial degeneration with elastin loss and fragmentation, further weakening the aortic wall.

**Table 1.** **Pathophysiology and Characteristics of TAA formation based on AV disease type**.

Mechanistic		AS-TAA		AI-TAA	
		TAV	BAV	TAV	BAV
		Altered blood flow/Hemodynamics		Abnormal leaflet coaptation	
				Stretched/Damaged cusps	
Clinical Patterns					
	Gender				Male predominance
	Age		Older		Young
	Morphology of aneurysm	Asymmetric	Asymmetric		Asymmetric
	Position of dilation		Tubular ascending aorta/ Eccentric		Aortic root (Annulus & SOV)
	Aortic dilation rate	Normal	Fast		Fastest
Aortic Valve Management		AVR	AVR	VSAR replacement if non-significant cusp disease (most common) or AVR (patient dependent)	AVR (most common) or VSAR replacement if adequate quantity and quality of leaflet tissue (increasing frequency & surgeon expertise dependent)
Post-AVR Course					
	Aortic dilation/aneurysm	Minimal/None	Lifelong-surveillance (root & ascending) despite AVR if no intervention on aorta at time of AVR		Lifelong-surveillance (root & ascending) despite AVR if no intervention on aorta at time of AVR
	AAE Risk	Minimal/None	Present		10× increase dissection risk even with AVR
Hemodynamic Changes					
	Peak systolic wall velocity		High in anterolateral region of aortic wall, elevated flow eccentrically		Elevated WSS

Abbreviations: AAE, adverse aortic event (dissection, rupture, death); AI, aortic insufficiency; AS, aortic stenosis; AVR, aortic valve replacement; BAV, bicuspid aortic valve; TAA, thoracic aortic aneurysm; TAV, tricuspid aortic valve; VSAR, valve sparing aortic root replacement; WSS, wall shear stress; AV, aortic valve; SOV, sinus of Valsalva.

**Table 2.** **AV and TAA Histopathology associated with type of AV Disease**.

	Aortic Stenosis (AS)	Aortic Regurgitation (AR)
Valve Structure	Thick nodular leaflets + fibrosis + calcification	Thin leaflets/Stiff
	Fibrosa rich in Collagen III	Fibrosa rich in Collagen I and III
	Low AGE	High diffused AGE/resistance to protease digestion
TAV-Aortas		Ascending aortic remodeling, severe medial degeneration, elastin loss and fragmentation, mucoid ECM accumulation
		Decreased fibrillin and collagen
		Decreased ENOS, subendothelial apoptosis
Evaluation in AVR patients	Medial degeneration and inflammatory markers	Increased medial degeneration and inflammatory markers (especially AR-TAV)
	Older AS-BAV patients	Younger AR-BAV patients
TAA- Aortas	EFL	Increased EFL (especially BAV patients & proximal aorta ≥50 mm)

Abbreviations: AI, aortic insufficiency; AGE, advanced glycation end products; AS, aortic stenosis; AV, aortic valve; AVR, aortic valve replacement; BAV, bicuspid aortic valve; ECM, extracellular matrix; EFL, elastic fiber loss; ENOS, endothelial nitric oxide synthase; TAA, thoracic aortic aneurysm; TAV, tricuspid aortic valves.

As such, AI patients (especially BAV-AI) should be followed more aggressively, both preoperatively and postoperatively following AVR for AI in the presence of mildly dilated proximal aortas. It is clear after analyzing all available data in the literature, that AI patients with aortopathy (dilation/aneurysms) represent a different risk group than those with AS or normal functioning AV. Unfortunately, with no current guidelines recognizing this special at-risk subgroup, these patients are improperly categorized into the general AV/TAA pathology population who are at lower risk of aortic dilatation and adverse aortic events.

Comprehensive aortic surveillance programs should not only include longitudinal anatomic analysis of the aortic root and ascending aorta via computed tomography (CT)/MRI scan, but also morphologic and functional analysis of the AV by echocardiography. Only then can we accurately perform risk assessments with fully informed data for these patients. Other non-invasive measures for improved assessments of aortic wall integrity should also be sought, with possible avenues of research to include biomarkers and improved imaging techniques.

A paradigm shift in the management of patients with AI irrespective of valve morphology is in order. Additional longitudinal research examining how the degree of AI impacts the risk of aortic dilatation and adverse aortic events will help strengthen this new framework and should be the first step. Longitudinal definition of the progression of AI with focus on the ascending aorta in BAV vs TAV will provide clearer guidelines for surgical intervention. Finally, further translational research will help identify the causes and pathways leading to TAA formation as a consequence of the distinct pathological AV phenotypes reported in this review.
